# Delayed Diagnosis of Posterior Urethral Valves in a 14-Year-Old Adolescent

**DOI:** 10.3390/reports9010076

**Published:** 2026-03-02

**Authors:** Christos Kyriopoulos, Anna Papakonstantinou, Evangelos Fragkiadis, Napoleon Moulavasilis, Panagiotis Mitsos

**Affiliations:** 1 Department of Urology, Agia Sofia Children’s Hospital, 11527 Athens, Greece; 2 First Department of Urology, Medical School, National and Kapodistrian University of Athens, 11527 Athens, Greece

**Keywords:** posterior urethral valves, vesicoureteral reflux, delayed diagnosis, adolescent

## Abstract

**Background and Clinical Significance**: Posterior urethral valves are rare congenital anomalies characterized by persistent urethral mucosal folds and causing varying degrees of obstruction. The increasing use of prenatal ultrasound has contributed to the early diagnosis of posterior urethral valves (PUV), a condition associated with a severe prognosis, as approximately one-fifth of affected patients develop chronic kidney disease. Early diagnosis and intervention—namely, valve ablation—provide obstruction alleviation and renal function preservation. Therefore, it is uncommon for patients to be diagnosed in adolescence or adulthood, when patients usually present with frequency, voiding dysfunction, daytime incontinence, enuresis, recurrent urinary tract infections, and renal insufficiency. **Case Presentation:** We present a 14-year-old adolescent with recurrent urinary tract infections. A thorough medical history, clinical examination, and standard imaging revealed bilateral vesicoureteral reflux and posterior urethral valves. The patient underwent urethrocystoscopy for diagnostic and therapeutic purposes and posterior urethral valve ablation. Despite these interventions, the persistence of his symptoms necessitated endoscopic correction of the vesicoureteral reflux and circumcision. During the 2-year follow-up, the patient remained asymptomatic. **Conclusions**: Symptomatic adolescent boys should undergo a standard urinary evaluation to identify undiagnosed congenital urinary tract diseases and maintain renal and bladder function.

## 1. Introduction and Clinical Significance

Posterior urethral valves (PUV) is a congenital obstructive deformity of the male urethra with an incidence rate of 1/5000–1/8000 live births [1,2]. It is the most frequent cause of lower urinary tract obstruction in male neonates. The diagnosis is suspected during a routine prenatal ultrasound. There is an increased incidence of perinatal mortality (2–7.5%) [3,4,5] and chronic kidney disease in patients suspected to have PUV before the 24th week of gestation [6]. PUV is considered a multifactorial condition; however, a recent study recognized three microduplications responsible for PUV formation in 155 patients with prenatally diagnosed lower urinary tract obstruction (LUTO) [7].

The main prenatal ultrasound findings suggestive of PUV are hydronephrosis, dilated ureters, megacystis with a thickened bladder wall, and the highly distinguishable keyhole sign, characterized by dilation and elongation of the posterior urethra. Oligo/anhydramnio is often present. The fetal kidneys produce urine as early as the 10th gestation week, and this urine is the main component of amniotic fluid by the second trimester. In PUV with impaired renal function, urine production may be reduced, thus contributing to minimal amniotic fluid and pulmonary hypoplasia [8]. Additionally, renal hyperechogenicity (a sign of renal dysplasia) and a perirenal urinoma may be present [9].

In selected patients, and after thorough informed consent of the parents, prenatal treatment for PUV is performed. Placement of a vesicoamniotic shunt is the most common treatment and aims to decompress the fetal urinary tract and prevent further bladder and kidney damage. However, a prospective, randomized multicenter study (PLUTO), despite limited recruitment, reported inconclusive results concerning long-term renal function in PUV patients and a high complication rate [10]. Another prenatal procedure proposed for PUV is valve ablation via fetal cystoscopy, which has high maternal and fetal complication rates [11].

Postnatally, the patient should undergo bladder drainage if PUV is suspected, even in the absence of VCUG confirmation. The treatment options for PUV include temporary urinary diversion (vesicostomy or ureterostomy) when the infant is premature or the urethra is too small to accommodate instrumentation, and valve ablation with a cold knife (the gold standard), resectoscope or laser [12].

The literature reports that 33–65% of PUV patients develop chronic kidney disease (CKD), whereas 14–25% develop end-stage kidney disease [12,13]. Nadir creatinine at the boy’s first birthday has been found to correlate with long-term renal function [14]. Wu et al. proposed different creatinine level cutoffs for individual risk of chronic renal disease [15]. Prognostic factors include prenatal signs, postnatal ultrasound parameters, and urinary and biochemical biomarkers. Klaus et al. suggested that the optimal predictor of progression to CKD was nadir creatinine level after valve ablation, whereas microalbuminuria was an insufficient biomarker, and further research was warranted [16,17]. Moreover, genetic polymorphisms related to the renin–angiotensin system, specifically those involving the angiotensin-converting enzyme (ACE) and the type II angiotensin receptor genes 1 and 2, are associated with worse renal function prognosis in PUV patients [18,19]. According to the study of Meneghesso et al., bladder function and nadir creatinine at 1 year of age are the most reliable predictors of deteriorating renal function in PUV patients [13].

Most cases of PUV are diagnosed in infancy and early childhood, usually during evaluation of prenatal hydronephrosis. Postnatal ultrasound confirms the presence of a non-emptying bladder, and voiding cystourethrography reveals the elongated and dilated posterior urethra and, often, vesicoureteral reflux (VUR) [20,21]. The incidence of VUR in patients with PUV ranges between 26% and 72%, whereas the prenatal finding of posterior urethral dilatation significantly raises the probability of postnatally diagnosed PUV vs. VUR [22,23]. Vesicoureteral reflux (VUR) is the most common cause of febrile urinary tract infections (UTIs) in children. Advancements in prenatal and postnatal care driven by the extensive use of ultrasound and other imaging modalities (MRI, VCUG) have significantly contributed to the timely diagnosis and treatment of the lower urinary tract, while defining anatomy and providing gross information on bladder and urethral function [24]. In PUV patients, VCUG reveals a trabeculated bladder with a thickened bladder wall and diverticula, often accompanied by VUR. A significant number of PUV-treated patients develop “valve bladder”, representing a broad spectrum of bladder dysfunction, manifesting years after definitive treatment.

In a few cases, PUV is diagnosed at an older age due to the wide spectrum of clinical symptoms of this congenital disease. Clinical manifestations, such as urinary incontinence, poor urinary stream, recurrent urinary tract infections, and nocturnal enuresis in older boys, should include investigations for PUV [25,26]. Therefore, in the literature, the delayed presentation and diagnosis of adolescent or adult PUV cases are rarely reported. Here we report a 14-year-old adolescent with autism who presented with recurrent UTIs, normal renal function and poor urinary stream due to undiagnosed PUV.

## 2. Case Presentation

**Figure 1:**
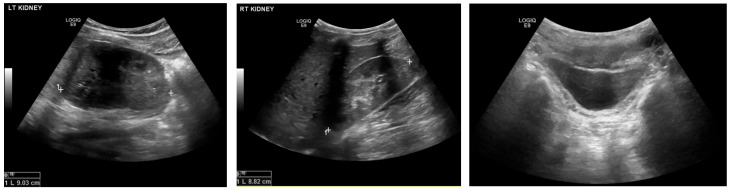
Ultrasound of his kidneys and bladder.

**Figure 2:**
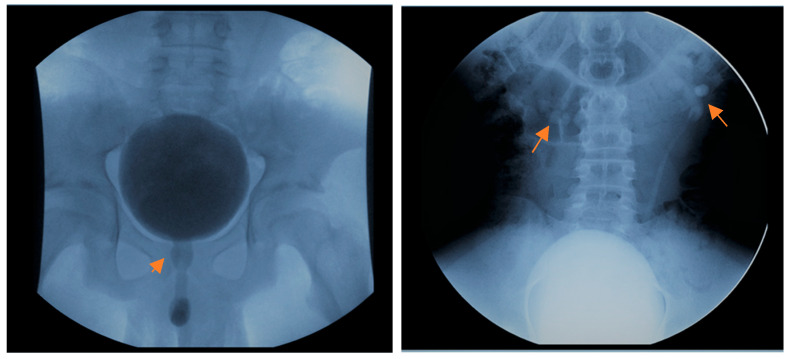
Dilatation and elongation of the posterior urethra, presence of bilateral VUR.

A 14-year-old adolescent presented to the outpatient department of the Urology Clinic with a normal antenatal history and three hospitalizations for recurrent febrile UTIs in the last year. The patient’s thorough clinical history was obtained, and he reported a thin stream of urination. Also, the young patient had an autistic spectrum disorder and a minor intellectual disability and was particularly difficult to cooperate with. Clinical examinations, including neurologic exam and lumbosacral spine palpation, were non-significant. Furthermore, the uroflowmetry showed an obstructive urinary curve. His renal function was normal (0.67 mg/dL). The ultrasound of the urinary tract was without significant pathological findings (Figure 1). At the same time, the voiding cystourethrography (VCUG) showed bilateral 2nd-3rd grade VUR, a normal-shaped bladder with absence of trabeculation, and mild dilatation of the posterior urethra (Figure 2). The DMSA renal scan showed pyelonephritic lesions in the right kidney.

**Figure 3:**
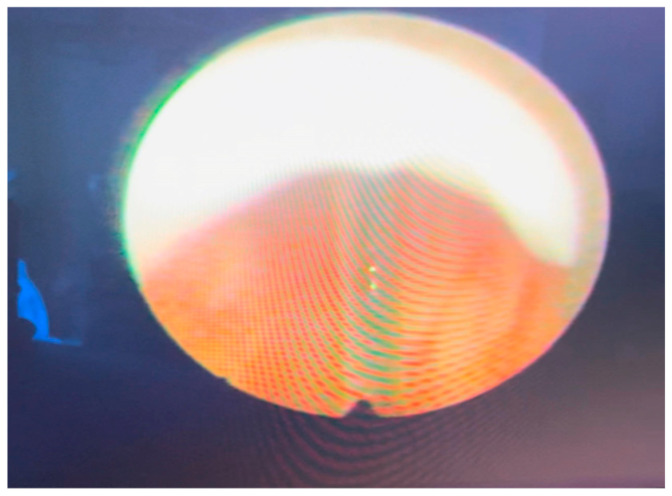
PUVs type 2 and cold knife ablation.

Based on these findings, the patient underwent urethrocystoscopy, which revealed mild bladder trabeculation, adequate bladder capacity, and bilateral orthotopic ureteric orifices. At the height of the verumontanum, we detected intraluminal projections (flaps), which were cranially projected and were classified as type 2 PUV according to Young’s classification (Figure 3). Then we performed an ablation of the posterior urethral valves using a cold knife.

Finally, during the Credé maneuver, the urination stream’s velocity and volume flow rate showed a clear improvement. The patient was uneventfully discharged with antibiotic prophylaxis. After his follow-up 3 months later, antibiotic prophylaxis was ceased and he was advised on regular bladder and bowel emptying. Two months later he presented with a new febrile UTI episode. The patient then underwent another imaging study with VCUG to assess for residual valves and the status of his vesicoureteral reflux. There was a noticeable improvement of the posterior urethra, but bilateral VUR remained, albeit with a grade reduction on the right side (Grade 1). Therefore, he underwent endoscopic correction of his VUR and circumcision in the same session. Since then, the patient has been followed up at 3, 6, and 12 months (per protocol), has remained asymptomatic without antibiotic prophylaxis, and his follow-up US and creatinine measurements were normal. Urodynamic studies were not performed due to the aggressive and uncooperative behavior of the patient. His father and the child were repeatedly informed of normal bladder and bowel function and optimal voiding habits.

## 3. Discussion

PUV is the most frequent cause of congenital bladder outlet obstruction. The severity of the PUV-caused obstruction varies from oligohydramnio and the consequential pulmonary hypoplasia, causing infant death, in severe PUV to less severe cases with minor voiding dysfunction and recurrent urinary tract infections in adolescence and adulthood. Today, prenatal ultrasound and early fetal intervention have contributed to early detection and a significant decrease in mortality rate in PUV patients [27]. A multidisciplinary postnatal approach (urologist, nephrologist, urotherapist) is considered the standard of care for PUV patients.

However, delayed presentation of PUV has been associated with voiding dysfunction (57%) [28], infertility due to anejaculation [29], and worsening renal function [13]. Vasconcelos et al. did not report any meaningful differences in long-term renal function outcomes between prenatally and postnatally diagnosed PUV patients. Nonetheless, a significantly increased incidence of symptomatic urinary tract infections was also observed in the postnatal PUV diagnosis group [4]. By contrast, Shields et al. demonstrated that patients with a late PUV diagnosis have worse outcomes concerning renal function [30]. A study in Finland with 193 patients who were followed up for 31 years showed that early presentation, elevated serum creatinine, bilateral VUR and recurrent UTIs correlated significantly with ESRD (22.8%) [31]. The broad spectrum of PUV severity could explain the conflicting results of these studies, as well as the normal renal function of our patient, despite the late diagnosis and treatment of PUV.

Patients with moderate-to-severe forms of PUV benefit from early diagnosis and even antenatal treatment with a vesicoamniotic shunt [32]. In comparison, patients with milder forms of PUV may present later in life with lower urinary tract symptoms and recurrent urinary tract infections [33]. In all cases, PUV patients may later develop progressive bladder dysfunction and worsening renal function, and that is why they require long-term follow-up. Implementing a standardized clinical pathway indeed improves short-term outcomes in PUV patients, as reported in the study by Rickard et al. [34]. A recent review highlighted that PUV patients may also exhibit complications later in their lives because of their congenital malformation, thus underlining the need for lifelong care [35]. The literature reports the late presentation of PUV in adolescents and young adults [26,36].

VUR affects approximately 1–3 out of 4 individuals with PUV and is easily differentially diagnosed early [22,23] with comprehensive imaging, including ultrasound and VCUG. Consequently, PUV treatment may be delivered promptly [21]. The majority of patients diagnosed later in childhood and adolescence present with mild symptoms and are diagnosed through clinical investigation, when medical assistance is sought for urinary frequency (59%), daytime incontinence and enuresis (47%), and history of recurrent UTIs (41%) [37,38]. Bilateral VUR is associated with poor overall kidney function in PUV patients [22,39]. This was not the case with our patient, who had normal kidney function, albeit with pyelonephritic lesions on his right kidney.

Clinicians should include PUV in the differential diagnosis of boys older than 5 years with recurrent UTIs, voiding dysfunction, and diurnal enuresis. According to the literature, these patients should undergo VCUG and, if there is ambiguity, urethrocystoscopy to evaluate for possible urethral involvement [37,38]. Daytime incontinence refractory to urotherapy and pharmacotherapy showed improvement in 80% of mild-to-moderate PUV patients after valve ablation, but not in dysfunctional voiders, according to a report by Nakai et al. [25]. A recent study in Australia, which included school-aged boys (4–16 years old) with absent renal or spinal pathology who were referred due to bladder dysfunction to pediatric urology outpatient clinics, identified PUV as the underlying cause in 32.8% [40].

Our patient presented with recurrent UTIs, normal renal function and a thin voiding stream and underwent VCUG, which revealed a mild case of PUV. Although type 2 valves are rare, considered nonobstructive, and subject to overclassification [25,41], our patient underwent transurethral valve ablation with significant improvement. He was then discharged with instructions for bladder and bowel management and without antibiotic chemoprophylaxis. However, the persistence of his symptoms (UTI) necessitated the endoscopic correction of the VUR and circumcision. A study from John Hopkins Medical Institution and Children’s Hospital Medical Center in Tehran, Iran, showed that valve ablation in PUV patients led to VUR resolution in 66.1% of patients [42]. Another study by Michel et al. showed that uncircumcised boys with PUV who also had high-grade VUR were at higher risk of a febrile UTI than those without VUR. This risk decreased at specific time points and particularly after 3 and 9 months of age [43].

## 4. Conclusions

Recurrent episodes of febrile UTI or bladder dysfunction in school-aged boys, despite a normal previous individual clinical history, mandate standard evaluation of their urinary tract, often revealing undiagnosed or neglected congenital diseases. Timely diagnosis and treatment are essential for preserving the patient’s renal and bladder function. If an urethral lesion is suspected, further investigations with VCUG and urethrocystoscopy are warranted.

## Data Availability

The original data presented in this study are included in the article, further inquired can be directed to the corresponding author.

## Abbreviations

The following abbreviations are used in this manuscript:

DMSA	Dimercaptosuccinic Acid
PUV	Posterior Urethral Valves
US	Ultrasound
UTI	Urinary Tract Infection
VCUG	Voiding Cystourethrogram
VUR	Vesicoureteral Reflux
